# A central hub model for remote delivery of multisite clinical trial interventions

**DOI:** 10.1371/journal.pdig.0001818

**Published:** 2026-11-11

**Authors:** Leigh Charvet, Giuseppina Pilloni, Jacqueline H. Becker, Juan Wisnivesky, Alex D. Federman, Shayna Pehel, Timothy S. Ko, Deborah C. Koltai, David S. Knopman, Daniel T. Laskowitz, Ashley O’Steen, Melissa James, Wendy Cox, Jeannie Karwowski, Kanecia O. Zimmerman

**Affiliations:** 1 Department of Neurology, NYU Grossman School of Medicine, New York, New York, United States of America; 2 Division of General Internal Medicine, Icahn School of Medicine at Mount Sinai, New York, New York, United States of America; 3 Department of Neurology, Duke University School of Medicine, Durham, North Carolina, United States of America; 4 Department of Neurology, Mayo Clinic, Rochester, Minnesota, United States of America; 5 Duke Clinical Research Institute, Duke University School of Medicine, Durham, North Carolina, United States of America; 6 RECOVER Patient, Caregiver, or Community Representative, Durham, North Carolina, United States of America; 7 Department of Pediatrics, Duke University School of Medicine, Durham, North Carolina, United States of America; Peng Cheng Laboratory, CHINA

## Abstract

Digital health technologies enable remote delivery of clinical interventions, creating opportunities to redesign multisite trials. Traditional trials require participating sites to deliver interventions independently, potentially increasing implementation variability and operational complexity. Centralized models instead allow local recruitment and assessments while coordinating intervention delivery through one hub. We describe the feasibility of this model in RECOVER-NEURO, an NIH-funded, five-arm phase 2 randomized trial overseen by the Duke Clinical Research Institute. The trial enrolled 328 participants across 22 US sites with cognitive symptoms associated with long COVID. Sites conducted recruitment, consent, and clinical assessments, while an NYU Langone Health hub managed study kit preparation and distribution, device programming, onboarding, remote supervision, adherence monitoring, and equipment recovery. Participants were randomized to BrainHQ cognitive training alone; BrainHQ plus cognitive rehabilitation (PASC-CoRE), delivered via telehealth by Mount Sinai and Emory University investigators; BrainHQ plus active transcranial direct current stimulation (tDCS); BrainHQ plus sham tDCS; or non-adaptive computer-based puzzles and games as an active comparator. Interventions were delivered remotely over 10 weeks using home-based study kits. Participants completed a mean of 44.0 ± 10.9 sessions over 68.8 ± 11.3 days. Of 320 participants who received intervention, 260 (81.3%) were consistently adherent. The hub initiated intervention within 13.5 ± 6.1 days of first participant contact, supported up to 168 concurrently active participants, and achieved a 96.9% equipment return rate. The intervention phase was completed in 375 days. These findings demonstrate the feasibility of centralized remote delivery of digital and device-based interventions in a large multisite trial and may inform future decentralized or hybrid clinical trials.

Trial Registration

ClinicalTrials.gov Identifier: NCT05965739.

## Author summary

Clinical trials are commonly conducted across multiple hospitals or research centers, where each site independently delivers the intervention. Although this model supports broad recruitment, it can introduce differences in treatment implementation and increase logistical burdens. Digital health technologies now enable remote intervention delivery, creating opportunities for new clinical trial models. We describe how a centralized intervention hub coordinated remote delivery in the RECOVER-NEURO clinical trial, which enrolled 328 participants across 22 United States sites with cognitive symptoms associated with long COVID. Participating sites conducted recruitment, consent, and clinical assessments, while the hub coordinated intervention logistics including shipment of study equipment, participant onboarding, remote supervision, and adherence monitoring. The study evaluated several remote interventions, including digital cognitive training, cognitive rehabilitation delivered through telehealth, and home-based transcranial direct current stimulation. Participants completed most of their assigned intervention sessions, and overall adherence was high. The centralized hub coordinated intervention activities for many participants simultaneously using common procedures for onboarding, monitoring, and technical support, while achieving high equipment recovery rates. These findings suggest that centralized operational hubs can support coordination of remote interventions across multiple clinical sites and may inform future decentralized or hybrid trial designs.

## Introduction

Recent advances in digital health technologies have transformed how clinical interventions can be delivered and evaluated. Mobile and web-based telehealth platforms now allow interventions to be administered remotely, creating new opportunities to redesign the operational structure of multisite clinical trials [1]. These technologies make it possible to recruit participants across geographically distributed clinical sites while coordinating intervention delivery through centralized systems that maintain standardized procedures across participants.

Traditional multisite clinical trials typically require each participating site to independently implement and deliver study interventions. While this model supports broad recruitment, it can introduce variability in treatment implementation, require extensive training across sites, and increase operational complexity [2]. Differences in local resources, staffing, and experience with study procedures may further contribute to inconsistencies in intervention delivery. As clinical trials increasingly incorporate digital therapeutics and device-based interventions, operational models that combine distributed recruitment with coordinated remote intervention delivery warrant further evaluation.

Centralized intervention delivery models represent one such approach. In this framework, recruitment and clinical assessments remain distributed across participating study sites, while intervention delivery is coordinated through a single national hub. This model allows standardized intervention procedures, centralized monitoring of adherence and safety, and direct technical support for participants. Centralization may also reduce the logistical burden on individual sites and enable more efficient coordination of complex interventions that require specialized equipment or monitoring. In addition, remote delivery may expand access for participants who face geographic, mobility, or scheduling barriers that can limit participation in traditional site-based trials.

Despite the increasing availability of telehealth technologies, relatively few large-scale randomized trials have described the operational infrastructure required to implement centralized remote intervention delivery across multiple sites, particularly when trials involve device-based treatments such as wearable neuromodulation technologies. Remotely delivered cognitive training interventions have demonstrated feasibility and potential cognitive benefits in neurological populations, including individuals with mild cognitive impairment and multiple sclerosis [3,4]. Structured cognitive rehabilitation approaches based on goal management and attention regulation strategies have also shown promise in improving executive functioning in clinical populations [5]. In addition, remotely supervised transcranial direct current stimulation (tDCS) protocols have demonstrated safety and feasibility when administered in home settings under centralized supervision [6].

The RECOVER-NEURO randomized clinical trial was designed to evaluate nonpharmacological interventions for cognitive symptoms associated with long COVID [7]. Long COVID affects an estimated 7% of adults in the United States and frequently includes persistent cognitive symptoms such as impaired attention, memory difficulties, and slowed processing speed [8,9]. The RECOVER-NEURO trial enrolled 328 participants across 22 clinical sites in the United States and evaluated three intervention approaches—digital cognitive training, structured cognitive rehabilitation, and transcranial direct current stimulation delivered in combination with cognitive training—across five randomized study arms [7,10]. The trial used a centralized intervention delivery model in which recruitment and clinical assessments were conducted at participating sites while intervention delivery was coordinated through a national hub located at NYU Langone Health. Detailed descriptions of the trial design have been reported previously, and primary clinical outcomes from the RECOVER-NEURO randomized clinical trial have been published elsewhere [7,10].

The present manuscript specifically describes the operational infrastructure and feasibility of the centralized intervention hub established at NYU Langone Health to coordinate remote intervention delivery across all participating sites. Understanding the operational requirements for implementing centralized intervention delivery systems across multisite trials is increasingly important as clinical trials incorporate digital therapeutics, remote monitoring technologies, and home-based device interventions.

## Methods

### Study design

RECOVER-NEURO was an NIH-funded, multicenter, five-arm phase 2 randomized clinical trial designed to evaluate nonpharmacological interventions for cognitive symptoms associated with long COVID [7]. Overall trial oversight and coordination were provided by the Duke Clinical Research Institute (DCRI). The trial enrolled 328 participants across 22 clinical sites in the United States (Fig 1). The RECOVER-NEURO trial was approved by Advarra Institutional Review Board (Protocol/approval number: Pro00070287) and by the institutional review board or ethics committee at each participating institution, as required. All participants provided written informed consent before study participation. The RECOVER-NEURO trial was registered at ClinicalTrials.gov (Identifier: NCT05965739; https://clinicaltrials.gov/study/NCT05965739). The study protocol is available in S1 Text.

**Fig 1:**
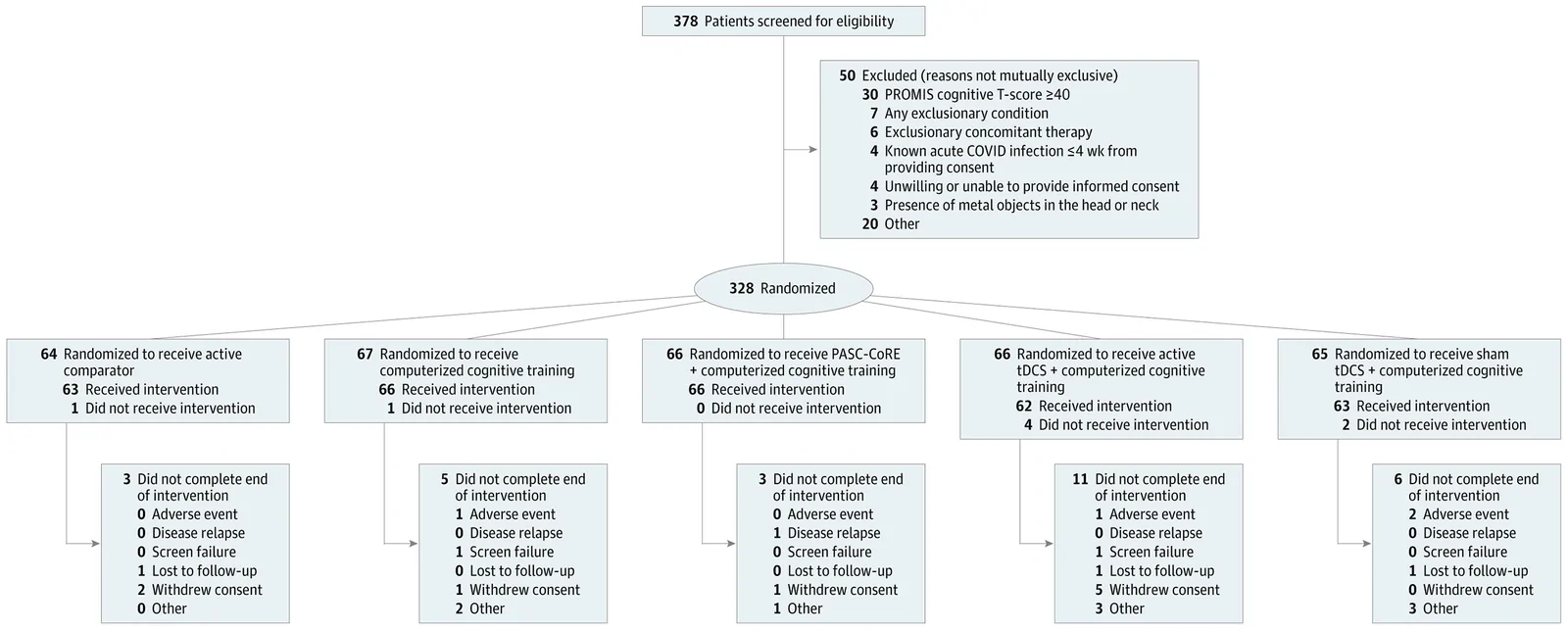
CONSORT flow diagram for the RECOVER-NEURO randomized clinical trial. The diagram summarizes screening, randomization, intervention receipt, and end-of-intervention completion across the five randomized study arms. The present manuscript reports operational and implementation outcomes related to centralized remote intervention delivery; primary efficacy and safety outcomes have been reported elsewhere.

Recruitment, eligibility determination, informed consent, and clinical outcome assessments were conducted locally at participating study sites. The centralized intervention hub at NYU Langone Health was responsible for operational implementation of the study interventions, including preparation and distribution of study kits, device programming for neuromodulation arms, participant onboarding, remote supervision of intervention sessions, monitoring of intervention adherence, and recovery of study equipment following completion of the intervention period (Fig 2).

**Fig 2:**
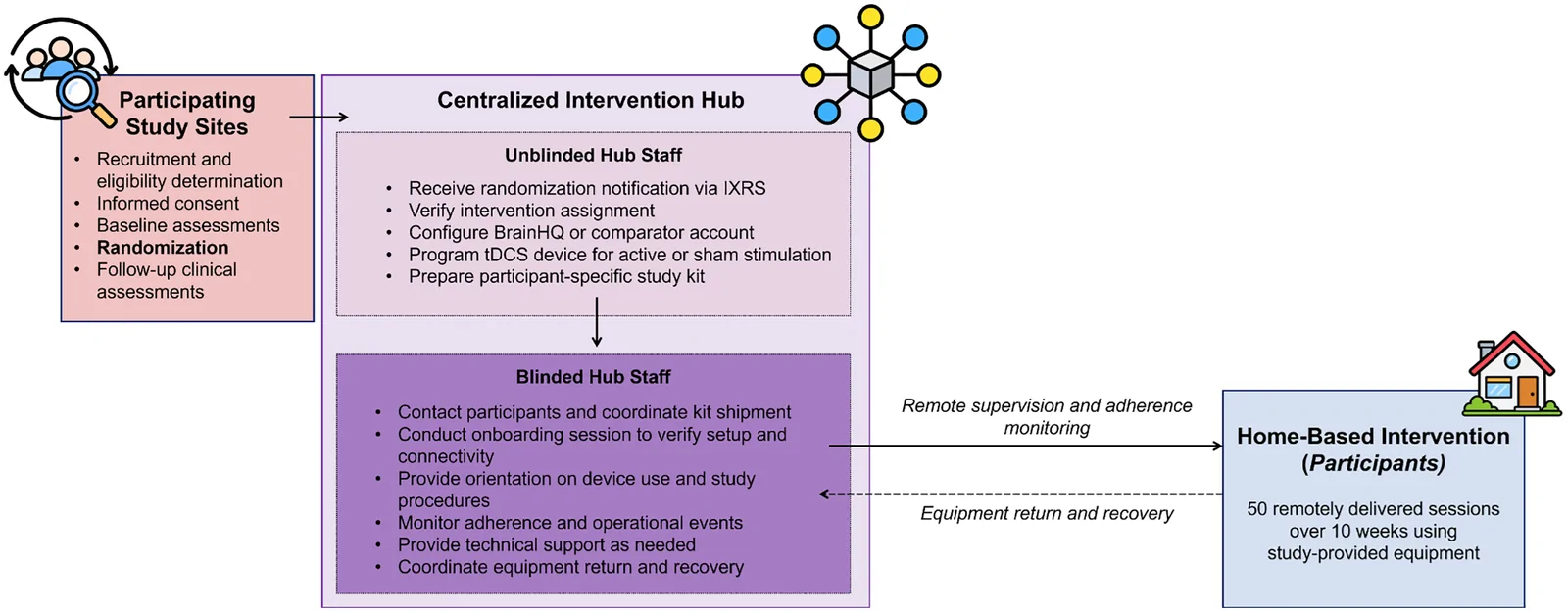
Centralized operational workflow for RECOVER-NEURO remote intervention delivery. Participating study sites conducted recruitment, eligibility determination, informed consent, baseline assessments, randomization, and follow-up clinical assessments. Following randomization, the centralized intervention hub coordinated participant-specific kit preparation, BrainHQ or comparator account configuration, tDCS device programming for active or sham stimulation, kit shipment, participant onboarding, remote supervision, adherence monitoring, technical support, and equipment recovery. Participants were prescribed 50 remotely delivered intervention sessions over 10 weeks using study-provided equipment. The centralized hub model supported standardized intervention delivery across 22 participating clinical sites.

Participants were randomized to one of five intervention conditions: (1) BrainHQ adaptive computerized cognitive training; (2) BrainHQ combined with cognitive rehabilitation (PASC-CoRE); (3) BrainHQ combined with active transcranial direct current stimulation (tDCS); (4) BrainHQ combined with sham tDCS; or (5) an active comparator consisting of non-adaptive computer-based puzzles and games. All interventions were delivered remotely over a 10-week intervention period.

Participants assigned to the PASC-CoRE arm received cognitive rehabilitation through telehealth sessions developed by investigators at Mount Sinai and delivered by investigators at Mount Sinai and Emory University. The centralized intervention hub at NYU Langone Health coordinated operational activities across all study sites, including study kit distribution, participant onboarding, adherence monitoring, and technical support, while directly managing the delivery of digital cognitive training (BrainHQ) and remotely supervised neuromodulation (tDCS).

Detailed descriptions of the overall trial design and clinical outcomes have been reported previously [7,10]. The present study focuses specifically on the operational infrastructure used to coordinate centralized remote intervention delivery across participating sites; the trial’s primary clinical efficacy outcomes have been reported elsewhere [7,10].

The centralized intervention hub was staffed by a core team of three personnel, with two staff members primarily responsible for day-to-day operations and a third providing technical support. Responsibilities included participant onboarding, device preparation, intervention supervision, adherence monitoring, technical troubleshooting, and equipment recovery. Two additional staff members provided temporary coverage during absences of core personnel to maintain continuity of operations. This staffing structure supported the coordination of interventions across all participating clinical sites and treatment arms. The core staffing structure remained unchanged throughout the intervention period, including during periods of peak participant activity.

### Randomization and workflow initiation

Following completion of baseline assessments at the enrolling site, eligible participants were randomized using the Interactive eXperience Response System (IXRS; Almac Clinical Technologies), a secure web-based platform used to manage treatment allocation within the trial infrastructure overseen by DCRI.

Once randomization occurred, the system generated the participant’s intervention assignment and automatically notified unblinded staff (who had access to participants’ treatment assignments) at the NYU Langone intervention hub. The notification included the participant study identification number and enrolling site. Blinded hub staff then accessed the participant record within the electronic data capture system (Medidata Rave) and initiated the operational workflow, including participant outreach, preparation of the participant-specific study kit, and scheduling of participant onboarding.

### Study kit preparation and distribution

Each participant received a home-based study kit assembled by the centralized intervention hub and shipped directly to the participant’s residence. The designated unblinded hub staff member programmed the devices of the intervention kit based on the assigned treatment arm.

Participants assigned to the tDCS intervention arms received a 1x1 mini-CT tDCS device (Soterix Medical, Woodbridge, NJ) preprogrammed by unblinded hub staff to deliver either active or sham stimulation according to the randomized assignment. The kit also included a headset (SNAPstrap) designed for standardized electrode placement over the dorsolateral prefrontal cortex and single-use saline sponge electrodes (SNAPpad) sufficient for the full intervention period. These participants additionally received an iPad tablet configured with the BrainHQ cognitive training platform (Posit Science) and teleconferencing software for communication with study staff.

Participants assigned to the BrainHQ-only, BrainHQ plus PASC-CoRE, or active comparator arms received an iPad tablet preconfigured with the appropriate BrainHQ training module or comparator program. Devices were also configured with teleconferencing software to enable remote communication with the intervention hub when needed.

Participants without reliable home internet access were provided with a mobile Wi-Fi hotspot with prepaid data coverage for the duration of the intervention period. All equipment was labeled with a unique serial number linked to the participant study identification number and shipped with prepaid return packaging.

### Participant onboarding and intervention initiation

After shipment of the study kit, hub staff contacted participants to confirm receipt and conduct an onboarding session. During this session, participants completed an unboxing video call with a study technician that included verification of device setup and connectivity, orientation and training on proper device use, and review of device safety features and study procedures prior to initiating the intervention.

The first three intervention sessions were conducted under direct supervision to ensure correct device use and familiarity with study procedures. Subsequent sessions were typically completed independently, with brief supervisory check-ins conducted when needed for technical support or adherence monitoring.

### Remote supervision of neuromodulation

Participants randomized to tDCS intervention arms completed stimulation sessions concurrently with cognitive training sessions. Prior to each stimulation session, participants connected briefly with a study technician located at the centralized intervention hub.

At the beginning of each session, participants provided a completion code generated by the tDCS device after the previous stimulation session. Study technicians verified the code to confirm that the prior stimulation session had been completed according to protocol. After verification, technicians issued a single-use unlock code that activated the device for the current stimulation session. This procedure enabled the centralized hub to monitor session completion and maintain safety oversight while allowing participants to complete stimulation sessions remotely in their home. Study technicians documented session completion, technical difficulties, and participant-reported concerns in daily session records, with potential safety concerns communicated to the enrolling clinical site according to study procedures.

### Monitoring of cognitive training adherence

Participants assigned to BrainHQ-based interventions completed cognitive training sessions independently following onboarding. Engagement with BrainHQ was monitored by the centralized hub through the BrainHQ clinician portal, which provided automated usage logs and timestamps of training activity.

Adherence metrics included total time spent on the platform and completion of scheduled training sessions. Deviations from expected engagement patterns prompted follow-up contact from hub staff to provide technical support or encourage continued participation. Adherence reports were also shared with participating study sites, which could independently follow up with participants regarding compliance or clinical concerns when appropriate. Participants assigned to the PASC-CoRE arm completed telehealth cognitive rehabilitation sessions delivered by collaborating investigators in parallel with BrainHQ training activities.

Daily technician records were also reviewed to identify platform access difficulties, software-related issues, connectivity problems, and technical support provided during cognitive training.

### Equipment return procedures

At the end of the 10-week intervention period, participants were instructed to return all study equipment using prepaid shipping materials included in the study kit. Participants could schedule a courier pickup arranged by the intervention hub or return the package to a designated shipping location. Returned equipment was logged and inspected upon receipt at the centralized hub. Devices were then reset and prepared for reuse when appropriate.

### Operational metrics and data analysis

The present study is a descriptive secondary analysis of operational data collected during the RECOVER-NEURO randomized clinical trial. Operational outcomes included intervention initiation timelines, session completion, adherence, concurrent participant activity, and equipment recovery. Data were obtained from centralized hub tracking records, intervention session logs, BrainHQ usage reports, and equipment shipment and return records.

Longitudinal adherence was defined as the proportion of scheduled intervention sessions completed during the intervention period. This threshold was part of the trial’s original weekly compliance-monitoring framework, in which adherence categories were determined by the number of weekly reports indicating completion of less than 80% of prescribed sessions. This approach was intended to capture sustained engagement and identify periods of reduced participation rather than relying solely on cumulative session completion. Participants were categorized as *Consistently Adherent* if no weeks fell below the 80% threshold. Participants with one to two weeks below the threshold were categorized as *Mostly Adherent*, those with three to four weeks below the threshold were categorized as *Sometimes Adherent*, and those with five or more weeks below the threshold were categorized as *Not Adherent*. Adherence analyses were restricted to participants who completed at least one intervention session. Participants who did not initiate treatment or had no recorded completed sessions were excluded from the adherence analysis but retained in the randomized population for participant flow reporting. Participants who discontinued intervention early were retained in the analysis and classified according to their available weekly adherence records. For participants assigned to active or sham tDCS, daily technician records were retrospectively reviewed to characterize stimulation delivery, technical support, and safety-related operational observations. Device- and code-related difficulties, electrode/contact problems, unsuccessful remote contacts, participant-reported sensations, and clinical consultations with the enrolling sites were summarized descriptively.

Operational timelines included the interval from randomization to first successful participant contact, from first contact to study kit shipment, and from first contact to initiation of the intervention. First contact was defined as the first successful communication between hub staff and the participant following randomization. Intervention initiation was defined as the first recorded intervention session. Equipment return time was calculated from the end of the intervention period to documented receipt of the returned study kit at the hub. Equipment recovery was defined as receipt of the study kit, based on return tracking records.

The number of participants simultaneously engaged in intervention activities was tracked over time to characterize the observed operational workload and maximum concurrent participant volume supported by the centralized hub. An active participant was defined as a participant whose intervention period had started and had not yet ended, based on recorded intervention start and end dates.

Descriptive statistics were used to summarize operational metrics. Continuous variables were summarized using means and standard deviations for approximately normally distributed data and medians with interquartile ranges when appropriate. Categorical variables were summarized using frequencies and percentages. Concurrent participant activity was plotted over time to visualize operational capacity during the trial.

## Results

### Participant enrollment and allocation

As described by Knopman et al (2026) [10], a total of 328 participants were enrolled in the RECOVER-NEURO trial across 22 participating clinical sites in the United States. Participants were randomized across five intervention arms as follows: 66 participants were assigned to BrainHQ combined with cognitive rehabilitation (PASC-CoRE); 67 participants to BrainHQ cognitive training alone, 66 participants to BrainHQ combined with active transcranial direct current stimulation (tDCS); 65 participants to BrainHQ combined with sham tDCS; and 64 participants to the active comparator condition consisting of non-adaptive computer-based puzzles and games (Fig 1).

Of the 328 randomized participants, 320 (97.6%) received at least one intervention session, while 28 did not complete the intervention, as reported in the CONSORT diagram.

### Operational capacity

The intervention period spanned 375 days, from September 9, 2023 to September 18, 2024. During this period, the centralized intervention hub coordinated delivery of interventions for participants enrolled across all study arms and participating sites. The centralized hub successfully contacted 327 of 328 participants following randomization. For the remaining participant, multiple contact attempts were made, but the participant could not be reached.

The number of participants actively engaged in the intervention increased progressively as recruitment expanded across sites (Fig 3), reaching an operational capacity peak of 168 participants simultaneously participating in intervention activities. At peak activity, 67 participants were engaged across the BrainHQ plus active and sham tDCS arms combined, 67 participants were engaged in either the BrainHQ-only or active comparator arms, and 34 participants were engaged in the BrainHQ plus PASC-CoRE arm (Fig 4).

**Fig 3:**
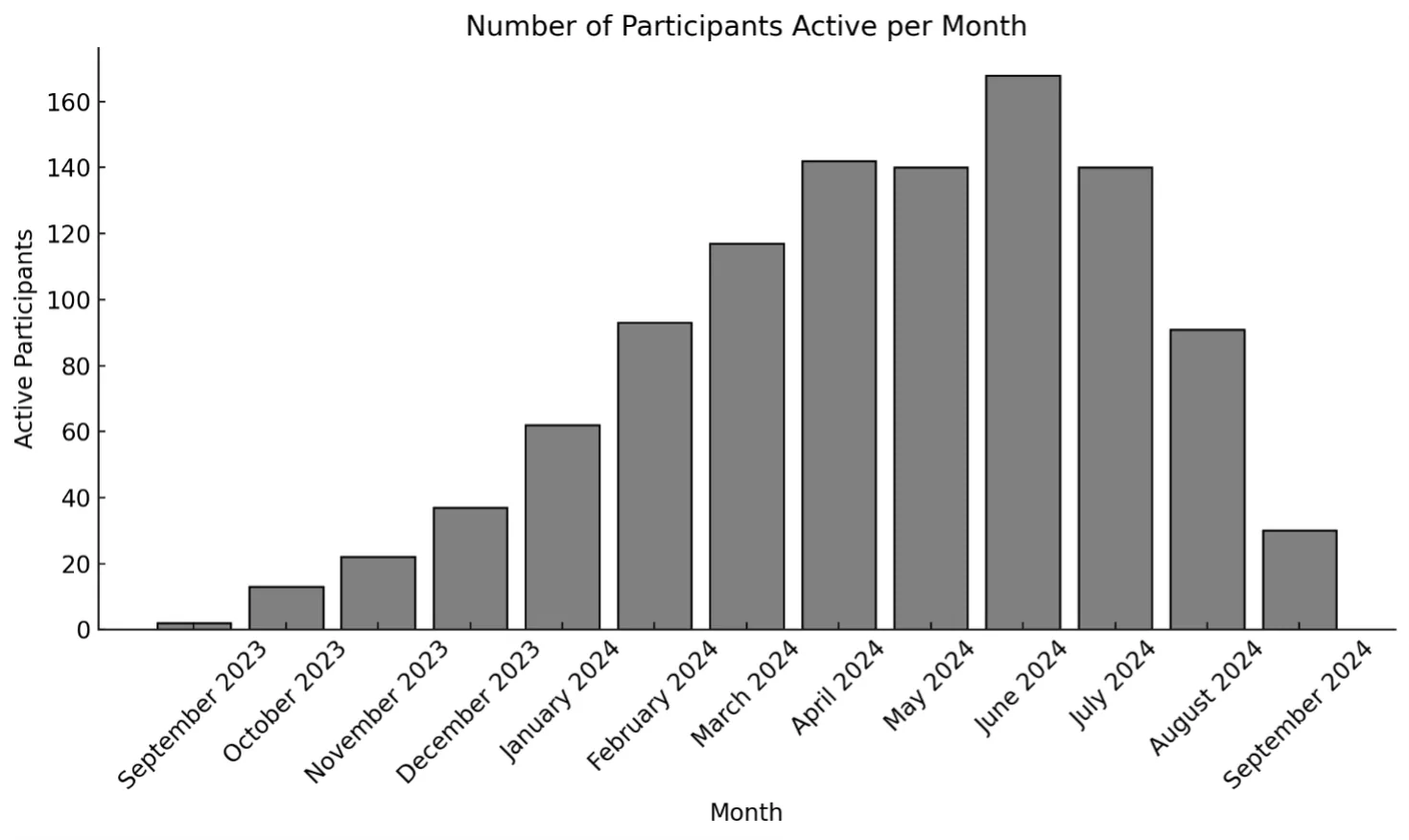
Monthly number of participants actively engaged in remote intervention delivery. The number of active participants increased as enrollment expanded across participating sites, demonstrating the centralized intervention hub’s capacity to support a growing remote intervention network. The hub reached a peak operational load of 168 concurrently active participants in June 2024.

**Fig 4:**
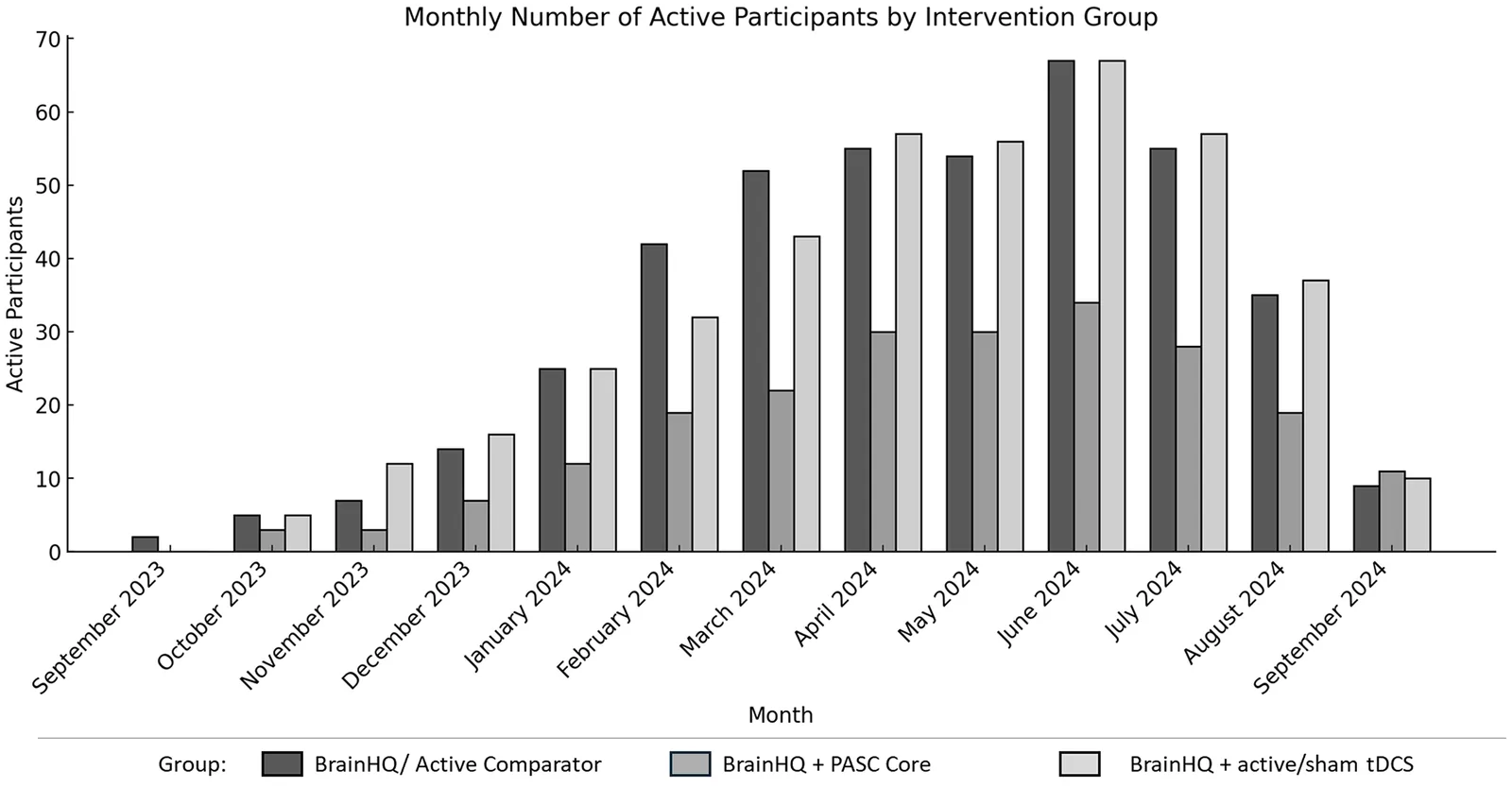
Monthly number of participants actively engaged by intervention group. Participant activity increased across all intervention groups as enrollment expanded, including BrainHQ or active comparator, BrainHQ plus PASC-CoRE, and BrainHQ plus active or sham tDCS. The figure shows the centralized intervention hub’s capacity to coordinate multiple remotely delivered intervention modalities concurrently over the course of the trial.

These findings indicate that the centralized hub coordinated multiple intervention modalities across 22 participating clinical sites, reaching a maximum concurrent workload of 168 active participants. The hub used common operational procedures across intervention arms, although implementation fidelity and between-site variability were not formally evaluated.

### Operational timelines

The centralized hub successfully contacted 327 of 328 participants following randomization; one participant was not contacted. The median time from randomization to first participant contact was 3 days (IQR: 1–6), with a mean of 4.0 ± 5.5 days (n = 326).

After initial contact, the median interval to intervention initiation was 13 days (IQR: 10–16), with a mean of 13.5 ± 6.1 days (n = 320). This interval included preparation of the participant-specific study kit, shipment of equipment to the participant’s residence, and completion of the onboarding process required for remote intervention delivery.

In some cases, participants elected to delay initiation of the intervention within the allowable study window to accommodate scheduling constraints. The mean time from initial participant contact to shipment of the study kit was 2.0 ± 0.5 days (range 1–3 days), reflecting the ability of the centralized hub to rapidly assemble and distribute intervention materials.

### Adherence and intervention completion

Participants completed a mean of 44.0 ± 10.9 intervention sessions over an average intervention duration of 68.8 ± 11.3 days.

Among the 320 participants who completed at least one intervention session, 260 (81.3%) were classified as *Consistently Adherent*, completing at least 80% of scheduled sessions during each assessed intervention week. Forty-one participants (12.8%) were classified as *Mostly Adherent*, with one to two weeks below the 80% adherence threshold. Thirteen participants (4.1%) were classified as *Sometimes Adherent*, with three to four weeks below the threshold, and six participants (1.9%) were classified as *Not Adherent*, with five or more weeks below the threshold. The complete adherence distribution for both analysis populations, including the eight participants who did not initiate intervention, is presented in **Table 1**. Longitudinal adherence monitoring also identified eight participants with three consecutive weeks of noncompliance. Participants who discontinued intervention early were retained in the analysis and classified according to their available weekly adherence records. Overall, adherence among participants who completed at least one intervention session was high. In a sensitivity analysis defining adherence as completion of at least 80% of the 50 prescribed sessions across the intervention period, 290 of 320 participants (90.6%) met the criterion, compared with 260 (81.3%) under the original weekly definition. Forty-eight participants (15.0%) were reclassified: 39 met the cumulative but not the weekly criterion, whereas nine met the weekly but not the cumulative criterion.

**Table 1:** Adherence classification by analysis population in the RECOVER-NEURO trial.

Adherence category	Received intervention (n = 320)	All randomized (n = 328)
Consistently Adherent	260 (81.3%)	260 (79.3%)
Mostly Adherent	41 (12.8%)	41 (12.5%)
Sometimes Adherent	13 (4.1%)	13 (4.0%)
Not Adherent	6 (1.9%)	6 (1.8%)
Did not initiate intervention	—	8 (2.4%)

These findings indicate that the majority of participants were able to complete most or all of the assigned intervention sessions using the centralized remote delivery model.

Operational outcomes stratified by intervention arm, including intervention initiation, session completion, adherence, intervention discontinuation, and equipment recovery, are summarized in **Table 2**.

**Table 2:** Operational outcomes by intervention arm.

Operational Metric	BrainHQ	BrainHQ + PASC-CoRE	BrainHQ + Active tDCS	BrainHQ + Sham tDCS	Active comparator
Randomized participants, N	67	66	66	65	64
Received intervention, n/N* (%)	66/67 (98.5%)	66/66 (100.0%)	62/66 (93.9%)	63/65 (96.9%)	63/64 (98.4%)
Time to intervention initiation, days, median (IQR)	12 (7–14)	19 (14–26)	12 (8.75–14.3)	13 (10–15)	12 (9–14)
Completed sessions, mean ± SD	43.7 ± 10.6	44.7 ± 7.8	42.0 ± 13.8	43.4 ± 12.8	46.0 ± 8.5
Consistently adherent, n/N^†^ (%)	53/66 (80.3%)	45/66 (68.2%)	51/62 (82.3%)	55/63 (87.3%)	56/63 (88.9%)
Did not complete intervention, n/N* (%)	5/67 (7.5%)	3/66 (4.5%)	11/66 (16.7%)	6/65 (9.2%)	3/64 (4.7%)
Study kits shipped, N	67	66	64	64	63
Equipment recovered, n/N^††^ (%)	63/67 (94.0%)	64/66 (97.0%)	63/64 (98.4%)	63/64 (98.4%)	61/63 (96.8%)

* Intervention receipt and intervention noncompletion percentages are based on randomized participants. † Adherence percentages are based on participants who received intervention. †† Equipment recovery percentages are based on study kits shipped.

### Safety monitoring and technical support

Across the active and sham tDCS arms, 5,508 intervention sessions were recorded (2,690 active and 2,818 sham). Daily technician records were available for 125 participants who initiated the active or sham tDCS intervention; n = 6 randomized participants did not initiate the active or sham tDCS intervention. Review of these records identified 71 substantive technical issue notes involving 53 participants, including 37 code-related difficulties (e.g., incorrect code entry, invalid codes, or session authorization difficulties), 29 device or power-related problems (primarily battery charging difficulties, low battery levels, or device shutdowns), and five electrode/contact issues. Safety-related session interruptions were not systematically quantified in the available technician records, and electrode-placement retraining events were not tracked as a separate operational metric. Eleven unsuccessful remote contacts were also documented. Two instances of additional clinical support were recorded: one consultation with the NYU Langone principal investigator regarding participant anxiety about tDCS and one recommendation to contact the enrolling site during a period of psychosocial distress. Participant-reported sensations included tingling, itching, and warmth, which are commonly associated with tDCS and were not treatment-limiting. One potentially tDCS-related adverse event, a minor skin reaction that resolved without significant sequelae, was reported through the trial’s clinical safety reporting.

For computerized cognitive training, daily technician records documented 12 platform access or login difficulties, 16 software-related issues, and 8 connectivity problems. Technical support included assistance with account access, troubleshooting software interruptions, and provision of internet connectivity support when needed.

### Logistics and equipment return

The centralized hub coordinated the preparation, distribution, and recovery of study equipment throughout the trial. Following completion of the intervention period, the median time to equipment return was 12.7 days (IQR: 7.6–23.7), with a mean of 22.3 ± 29.7 days. Although return timelines varied across participants, the overall equipment recovery rate was high. Of the 324 study kits shipped, 10 (3.1%) were not returned, primarily due to participant nonresponse or loss of contact.

These findings demonstrate the logistical feasibility of distributing and recovering study equipment within a large-scale home-based multisite clinical trial coordinated through a centralized operational hub.

## Discussion

The RECOVER-NEURO randomized clinical trial implemented a centralized operational hub to coordinate remote delivery of multiple interventions across a large multisite clinical trial network. In this trial, 328 participants were enrolled and assessed across 22 clinical sites in the United States while intervention delivery was coordinated through a centralized operational hub at NYU Langone Health. This model enabled coordinated implementation of several remotely delivered interventions including digital cognitive training, structured cognitive rehabilitation (PASC-CoRE), and home-based tDCS across geographically distributed sites using standardized procedures.

A defining feature of this operational model was the separation of participant recruitment and clinical assessment activities from intervention delivery operations. Participating clinical sites conducted screening, informed consent, and outcome assessments while overall trial oversight was provided by the Duke Clinical Research Institute. The centralized intervention hub coordinated operational aspects of intervention delivery including preparation and distribution of study kits, participant onboarding, neuromodulation supervision, adherence monitoring, and recovery of study equipment. This structure separated site-based clinical assessments from intervention operations and allowed the centralized hub to apply common procedures for device preparation, participant onboarding, adherence monitoring, and technical support across participating sites. The centralized infrastructure also supported delivery of multiple intervention modalities within a single operational framework. The hub coordinated five randomized study arms simultaneously, including digital cognitive training, cognitive rehabilitation delivered through telehealth, and device-based neuromodulation. At peak activity, the centralized hub coordinated intervention activities for 168 concurrently active participants, including 67 participants assigned to active or sham tDCS. This represents the maximum concurrent workload managed within the existing operational structure. Although these findings support the feasibility of centralized coordination across multiple sites and intervention modalities, they do not establish whether the model could accommodate further increases in participant volume without additional staffing, resources, or changes in operational performance.

Participant adherence further supports the feasibility of this approach. Participants completed an average of 44 intervention sessions during the 10-week intervention period and more than 80 percent of participants met criteria for consistent adherence with the intervention schedule. These findings are consistent with previous studies demonstrating that remotely delivered cognitive training and telerehabilitation interventions can achieve high levels of engagement when supported by structured remote supervision and technical support systems.

The centralized hub incorporated structured safety oversight for remotely supervised home-based tDCS. Device-generated completion codes and single-use unlock codes allowed technicians to monitor session completion and provide technical support as needed. Technician records documented technical difficulties, expected stimulation-related sensations, and two instances requiring additional clinical support. Common sensations, including tingling, itching, and warmth, were consistent with the established tolerability profile of tDCS. Only one event was formally reported as potentially related to tDCS, consisting of a minor skin reaction under an electrode that resolved without significant sequelae. These findings support the operational feasibility of integrating technical supervision and safety monitoring into a multisite clinical trial, consistent with previous reports of remotely supervised tDCS [6,11].

The operational experience of the RECOVER-NEURO trial demonstrates the feasibility of integrating multiple intervention modalities into a large-scale, multisite clinical trial infrastructure involving geographically distributed participants. Despite this complexity, the centralized hub coordinated interventions using common operational procedures and achieved high participant adherence and substantial concurrent operational capacity. Prior studies have shown that home-based cognitive training, telerehabilitation, and remotely supervised tDCS can be feasible [3,4,6], but many were single-center or focused on a single intervention modality. RECOVER-NEURO extends this work by integrating multiple remote intervention modalities within one multisite randomized trial, while separating local recruitment and outcome assessment from centralized intervention delivery [7,10]. This required the hub to coordinate onboarding, device preparation, remote supervision, adherence monitoring, technical support, and equipment recovery across 22 sites and five treatment arms. Together, these findings suggest that centralized operational hubs may provide a practical framework for coordinating digital therapeutics, telehealth-based behavioral interventions, and home-based medical devices across geographically distributed clinical trial sites. Beyond their potential operational advantages, centralized remote intervention models may also improve access to clinical trial participation for individuals who live far from major academic medical centers or who face geographic or mobility-related barriers to repeated in-person visits.

Primary clinical outcomes from the RECOVER-NEURO randomized clinical trial have been reported previously [10]. The present report focuses specifically on the operational systems that enabled centralized delivery of multiple remote interventions within a national trial network. Cost-effectiveness is an important consideration for determining the feasibility, resource requirements, and sustainability of centralized remote intervention delivery models in real-world settings. The present analysis was not designed to compare costs between centralized and site-based delivery, and detailed data on site-level staffing, training, infrastructure, and resource use were not collected for that purpose. Nevertheless, centralizing specialized functions such as device programming, participant onboarding, adherence monitoring, technical support, and equipment recovery may reduce duplication of effort across sites. Future studies should prospectively evaluate cost, staffing needs, and resource utilization as implementation outcomes.

### Limitations

Several limitations should be considered when interpreting these findings. First, the operational analysis is based on implementation within a single randomized clinical trial conducted among participants with cognitive symptoms associated with long COVID. Although the centralized intervention delivery model proved feasible within this context, additional studies will be needed to determine how well similar operational frameworks may generalize to other clinical populations, therapeutic areas, or intervention types. In considering real-world implementation of centralized remote intervention delivery models, generalizability may be limited by differences between clinical trial participants and broader clinical populations. Participants in this study were volunteers enrolled in a structured clinical trial and had sufficient access to technology and remote support to complete study procedures. Importantly, the feasibility observed in this trial was supported by dedicated operational resources, including provision of mobile hotspots and prepaid data plans for participants without reliable internet access. Thus, although interventions were delivered remotely, this model should not be considered inherently low-resource, and its implementation in other settings may require comparable technical, staffing, and logistical support.

Second, the centralized intervention delivery model was implemented as part of the RECOVER-NEURO randomized clinical trial, which was designed to evaluate clinical interventions rather than to formally compare centralized and site-based intervention delivery models. As a result, the operational performance of the centralized hub cannot be directly compared with traditional site-based delivery approaches.

Third, operational outcomes were evaluated descriptively within a single trial implementation, without a formal assessment of scalability or comparison with alternative delivery models. Although the centralized hub coordinated interventions for up to 168 concurrently active participants, this observation does not establish how staffing requirements, resource utilization, or operational performance would change with further increases in participant volume. Detailed measures of staff effort, technician-to-participant ratios, time spent per participant, technical support workload, and operational performance during periods of varying enrollment were not systematically evaluated. Future studies should prospectively assess these outcomes, together with operational costs and long-term sustainability, to determine the scalability and reproducibility of centralized intervention infrastructures. Additionally, although common operational procedures were implemented across participating sites, intervention fidelity, protocol deviations, and between-site variability were not systematically evaluated. While centralization may theoretically improve implementation consistency and operational efficiency, these potential benefits require further empirical evaluation.

Finally, the operational model relied on a centralized technical and staffing infrastructure responsible for coordinating intervention delivery across sites. Future studies should also include a broader range of participants, including those with higher levels of disability and those who are caregiver dependent. Replication of this model in other trials may require similar operational coordination and technical resources.

## Conclusion

This study demonstrates that a centralized operational hub can successfully coordinate standardized remote delivery of complex interventions within a large multisite randomized clinical trial. This operational framework enabled the simultaneous implementation of multiple remotely delivered interventions including digital cognitive training, telehealth-delivered cognitive rehabilitation, and home-based neuromodulation across geographically distributed sites using standardized procedures.

Despite the logistical complexity associated with coordinating multiple clinical sites, intervention modalities, and remotely delivered device-based treatments, the centralized hub supported intervention delivery across five randomized study arms and achieved a peak concurrent workload of 168 participants. These findings provide evidence of operational feasibility within the RECOVER-NEURO trial. Further evaluation of staffing requirements, resource utilization, and operational performance across varying participant volumes is needed to determine whether this model can be scaled efficiently and reproduced in other trial settings.

These findings suggest that centralized intervention delivery models may provide a practical operational framework for coordinating remotely delivered therapies across multisite clinical trials. As digital therapeutics, telehealth-based treatments, and home-based medical devices continue to emerge, centralized operational hubs may provide a practical framework for coordinating clinical trials and evaluating emerging interventions across geographically distributed populations.

## Supporting information

S1 Text Study protocol. (PDF)
